# Y-Like Retinal Ganglion Cells Innervate the Dorsal Raphe Nucleus in the Mongolian Gerbil (Meriones unguiculatus)

**DOI:** 10.1371/journal.pone.0018938

**Published:** 2011-04-28

**Authors:** Liju Luan, Chaoran Ren, Benson Wui-Man Lau, Jian Yang, Gary E. Pickard, Kwok-Fai So, Mingliang Pu

**Affiliations:** 1 Department of Anatomy, School of Basic Medical Sciences, Peking University, Beijing, China; 2 Key Laboratory on Machine Perception, Peking University, Beijing, China; 3 Key Laboratory for Visual Impairment and Restore, Peking University, Beijing, China; 4 Department of Anatomy, Research Center of Heart, Brain, Hormone and Healthy Aging, LKS Faculty of Medicine, and The State Key Laboratory of Brain and Cognitive Sciences, The University of Hong Kong, Pokfulam, Hong Kong, China; 5 School of Veterinary Medicine and Biomedical Sciences, University of Nebraska-Lincoln, Lincoln, Nebraska, United States of America; 6 Joint Laboratory for Brain Function and Health, Jinan University and The University of Hong Kong, Guangzhou, China; Dalhousie University, Canada

## Abstract

**Background:**

The dorsal raphe nucleus (DRN) of the mesencephalon is a complex multi-functional and multi-transmitter nucleus involved in a wide range of behavioral and physiological processes. The DRN receives a direct input from the retina. However little is known regarding the type of retinal ganglion cell (RGC) that innervates the DRN. We examined morphological characteristics and physiological properties of these DRN projecting ganglion cells.

**Methodology/Principal Findings:**

The Mongolian gerbils are highly visual rodents with a diurnal/crepuscular activity rhythm. It has been widely used as experimental animals of various studies including seasonal affective disorders and depression. Young adult gerbils were used in the present study. DRN-projecting RGCs were identified following retrograde tracer injection into the DRN, characterized physiologically by extracellular recording and morphologically after intracellular filling. The result shows that DRN-projecting RGCs exhibit morphological characteristics typical of alpha RGCs and physiological response properties of Y-cells. Melanopsin was not detected in these RGCs and they show no evidence of intrinsic photosensitivity.

**Conclusions/Significance:**

These findings suggest that RGCs with alpha-like morphology and Y-like physiology appear to perform a non-imaging forming function and thus may participate in the modulation of DRN activity which includes regulation of sleep and mood.

## Introduction

The dorsal raphe nucleus (DRN) of the mesencephalon is a complex multi-functional and multi-transmitter nucleus involved in a wide range of behavioral and physiological processes. Numerous studies demonstrate that the DRN receives a wide range of inputs including afferents from the locus coeruleus, the lateral habenula, several midbrain areas including the substantia nigra, and the periaqueductal gray, as well as fibers from the hypothalamus and the medial prefrontal cortex [1]. The retina sends axons to the DRN although little is known about this retinoraphe projection. Two early studies showed that the DRN received a direct retinal input, which consisted of a small number of retinal ganglion cells (RGCs), some of which exhibited alpha-like morphology [2], [3]. Fite and colleagues continued this line of work and reported a substantial number of DRN-projecting RGCs, with both small and large soma sizes [4], [5] and suggested that these cells arose from the non-image forming component of the retina [5]. Intrinsically photosensitive retinal ganglion cells (ipRGCs) are considered the primary retinal component mediating non-image forming functions and these cells project to various visual and non-visual nuclei including lateral geniculate nucleus (LGN), suprachiasmatic nucleus (SCN), intergeniculate leaflet (IGL) of the LGN complex, and olivary pretectal nucleus [6], [7], [8], [9], [10], [11], [12], [13]. There is no evidence that ipRGCs project to the DRN [14]. Therefore, determining the morphological and physiological properties of DRN-projecting RGCs will provide much needed information about the type of retinal information processing performed by the DRN.

Visual response properties of two major types of retinal ganglion cell were first described by Enroth-Cugell and Robson [15] and were classified as X and Y cells. Based on distinct morphological properties of two major retinal ganglion cell types, Boycott and Wässle named them as alpha and beta cells [16]. Taking physiological response properties into consideration, they suggested correlations between the morphological category alpha cells and the physiological class Y cells and also between beta cells and the X cells. Peichl and Wässle (1981) completed morphological identification of Y cells in the cat retina [17]. Over the last fifty years, the brisk-transient visual response pattern of Y-like cells has been confirmed in many species [18], [19], [20], [21], [22] but see Pang and coworkers [23]. It has been demonstrated that the Y (alpha)-like RGCs are preserved in every mammalian species examined [24]. In addition, available anatomical evidence reveals that the Y (alpha) cells only innervate visual related nuclei including the dorsal lateral geniculate nucleus, superior colliculus (SC) and pretectum [21], [25], [26], [27], [28], [29], [30], [31]. Therefore, all available evidence indicates that Y (alpha)-like cells are involved in processing visual rather than non-visual photic information.

Using intracellular filling and single-cell recording from retrogradely labeled DRN-projecting RGCs, we found that the vast majority of DRN-projecting ganglion cells exhibit classic alpha-like dendritic morphology and Y-like visual response pattern. These cells do not express detectable levels of melanopsin nor do they provide any evidence of being intrinsically photosensitive. Therefore, these DRN-projecting Y (alpha)-like cells appear to be responsible for processing non-visual information.

## Materials and Methods

### Animals

Female young adult Mongolian gerbils (Meriones unguiculatus ) were used in this experiment. Animals were housed in a 12-hour light∶12-hour dark cycle and food and water were provided ad libitum. All experiments were performed in accordance with Peking University guidelines for animal research and the Association for Research in Vision and Ophthalmology (ARVO) Statement for the Use of Animals in Ophthalmic and Vision Research. The experimental animal protocol was approved by Peking University Institutional Animal Care and Use Committee (Approval ID: SKXJ-2006-0025, effective 12/27/2006). The animals were anesthetized (60 mg/kg sodium pentobarbital, intraperitoneally) and placed in a stereotaxic instrument. After a craniotomy was performed, a Hamilton syringe was inserted and stereotaxically positioned in the DRN. In each penetration, 0.5 µl of fluorescent microspheres or 8% biotinylated dextran-conjugated methylrhodamine (Micro-ruby, Invitrogene Inc, Carlsbad, CA) was deposited in the right or left DRN. In some experiments, microspheres of a different color were delivered to the dLGN or SC to determine if DRN-projecting ganglion cells had axonal collaterals that also innervated the dLGN or SC. After tracer injections, the wound was sutured, and the animal was allowed to recover.

### In vitro preparation

The whole-mounted retinal preparation has been described previously [32]. Briefly, the animal was dark adapted for 40 minutes before enucleation and under dim red light, the lens and vitreous were carefully removed with a pair of fine-forceps. The eyecup was flat mounted, sclera side down, directly on the bottom of a recording chamber and was superfused by oxygenated (95% O^2^/5% CO^2^) Ames medium (Sigma-Aldrich, St. Louis, MO) at a fixed rate (5 ml/min) at room temperature between 22–24°C.

### Visual stimulation

Computer generated visual stimulation paradigms have been described previously [33]. Briefly, visual stimuli were generated by programming the Psychophysics Toolbox in Matlab [34] displayed on a Samsung mini LED projector (Samsung SP-P310ME, Samsung Electronics Co Ltd, Suwon City, Korea) and imaged with a first-surface mirror and lens (Edmond Scientific, Barrington, NJ) on the film plane of the microscope's camera port. The luminance level of the projector was measured with a digital radiometer (S370 Radiometer, UDT Instruments, San Diego, CA) using a 40× water-immersion objective (Carl Zeiss., Thornwood, NY). With this objective and a testing spot (750 pixels in diameter), the maximum luminance was 580 cd/m**^2^**on the retinal surface and the background was 47 cd/m**^2^**), based on the assumption that, at 500 nm, 1 cd/m**^2^** = 1 lumen/m**^2^** = 1/683×25.15×10**^17^/**m**^2^**/s = 3.68×10**^15^**/m**^2^**/s = 3.68×10**^11^** photons/cm**^2^**/s. Therefore, the unattenuated maximum irradiance from the projector was equal to 580×3.68×10**^11^** photons/cm**^2^**/s = 2.13×10**^14^** photons/cm**^2^**/s. The irradiance was further reduced by using neutral density filters (Oriel Corp., Stratford, CT). The details of the linearity test are described elsewhere [32]. Briefly, stimuli used were temporally counter phase modulated sinusoidal gratings of various spatial frequencies (0.08, 0.1, or 0.11 cyc/deg). Temporal frequency was 2 Hz. The spatial phase of the grating was advanced from 0° to 360° across the receptive field in 30° increments. The wavelength of the stimulation was produced by using a 475 nm narrow band interference filter (Oriel Corp., Stratford, CT). The maximum intensity of the irradiance that this 475 nm light could reach was 4.3×10**^14^** photons/cm^2^/s. The intensity of the output was attenuated by a set of neutral density filters.

### Physiological recording and data analysis

Visual responses were recorded extracellularly using a glass microelectrode and amplified with a patch clamp amplifier (Multiclamp 700B) and digitized (Digidata 1430; Axon Instrument, Inc., Forest City, CA). RGCs that accumulated fluorescent microspheres were selected for recording. The receptive field was mapped with a 0.2° test spot. An area-threshold test was then carried out to determine the best spot size that evoked the maximum discharge. After 40 minutes of dark adaptation, the optimized spot size was selected for the increment luminance threshold test. The intensity of background and visual stimulation was programmed and attenuated sufficient to maintain the retinas in the completely dark-adapted state. The tests were carried out following previously published methods [33]. To test the linearity of a recorded cell, we first plotted post-stimulus time histograms (PSTH) of the cell at difference spatial phases. For Y cells with nonlinear spatial summation, this modulation would occur mainly at twice the temporal modulation frequency (frequency doubling). Next, conventional Fourier analysis techniques were performed to determine the amplitude of response components at the frequency of stimulation (fundamental) and at the second harmonic. The acquired data were further analyzed off-line (pCLAMP9; Axon Corp., CA).

### Drug application

The synaptic blocker cocktail consisted of a mixture of excitatory and inhibitory neurotransmitter blockers including L-(+)-2-Amino-4-phosphonobutyric acid (AP-4, 100 µM), 6,7-Dinitroquinoxaline-2,3-dione (DNQX, 20 µM), DL-2-amino-5-phosphonovaleric acid (AP5, 50 µM), Picrotoxin (50 µM ) Strychnine (0.3 µM), and hexamethonimm bromide (200 µM) was added to the oxygenated Ames' medium (Sigma Chemical Co, St Louis, MO). In early experiments, cobalt chloride (2 mM) was added alone or included in the mixture. This solution was delivered to the recording chamber via a dispensing pump (Reglo, ISMATEC SA, Glattbrugg, Switzerland) at 5 ml/min and the response patterns of RGCs were recorded before, during, and after drug application.

### Intracellular staining

After survival times of 3–5 days, the animal was anesthetized as above, and the eye was enucleated and hemisected. The retina was carefully separated from the eyecup and flat-mounted on a filter paper (GS, 0.22 mm, Millipore Corp., Bedford, MA) with the ganglion cell layer facing up. The retina was placed in an injection chamber and superfused with oxygenated Ames' medium continually at a fixed rate (3 ml/min). Retrogradely labeled cells were filled using a microelectrode containing 1% Microruby (Invitrogen Inc, Carlsbad, CA) and 3% neurobiotin (Vector Laboratories, Burlingame, CA). A small amount of current (1–2 nA for 1–2 min) was applied to the electrode until the soma and process terminals were completely stained.

### Immunohistochemistry and image processes

The labeled cells were double stained with anti-melanopsin and anti-rhodamine antibodies. Details of the staining techniques were described elsewhere [34]. Briefly, the retinas were placed in 10% goat serum in 0.3% Triton-X-100 and phosphate-buffered saline mixture (Vector Laboratories Inc, Burlingame, CA) for 1 h before incubating in primary antibodies: anti-melanopsin polyclonal antibody (PT1-780, 1∶500, Fisher Scientific, Pittsburgh, PA) and biotinylated anti-rhodamine (1∶100, Vector Laboratories) for 36 h at room temperature. The retinas were then placed in secondary antibodies including Alexa Fluor 488-conjugated IgG (1∶500, Invitrogen Inc, Carlsbad, CA) and Cy5- conjugated streptavidin (1∶1000, Jackson Immuno-Research, West Grove, PA) for 2 h at room temperature. All retinas were rinsed and cover-slipped with the ganglion cell side up in aqueous mounting medium (Dako Corp., Carpinteria, CA). Ganglion cells were scanned with a confocal microscope (TCS SP5 II, Leica Microsystems, Wetzlar, Germany). The Z-axis interval was 0.2 µm. Each stack of optical sections covered a retinal area of 325.75×325.75 mm2 (1024×1024 pixels). By using Image J and Photoshop CS5 (Adobe Corp., San Jose, California, USA), each stack of optical sections were montaged and projected to a 0° X–Y plane and a 90° Y–Z plane to obtain a three-dimensional reconstruction of the cell. Details of three-dimensional reconstruction and confocal calibration procedures were described elsewhere [35].

### Injection site verification

After enucleation, the animals were sacrificed with an overdose of Nembutal and perfused with physiological saline followed by 4% paraformaldehyde. After postfixation with 4% paraformaldehyde, the brain was sectioned with a cryostat microtome (CM1900, Leica Microsystems, Bannockburn, IL) at 50 µm per section in the coronal plane from the anterior to posterior mesencephalon covering the whole length of the DRN. The sections were mounted on slides and examined under the fluorescence microscope (Zeiss Axioskop 40, Carl Zeiss, Thornwood, NY) to verify the injection sites and to evaluate whether injections had invaded other visual related nuclei.

## Results

### Dendritic morphology of DRN-projecting ganglion cells

**Figure 1:**
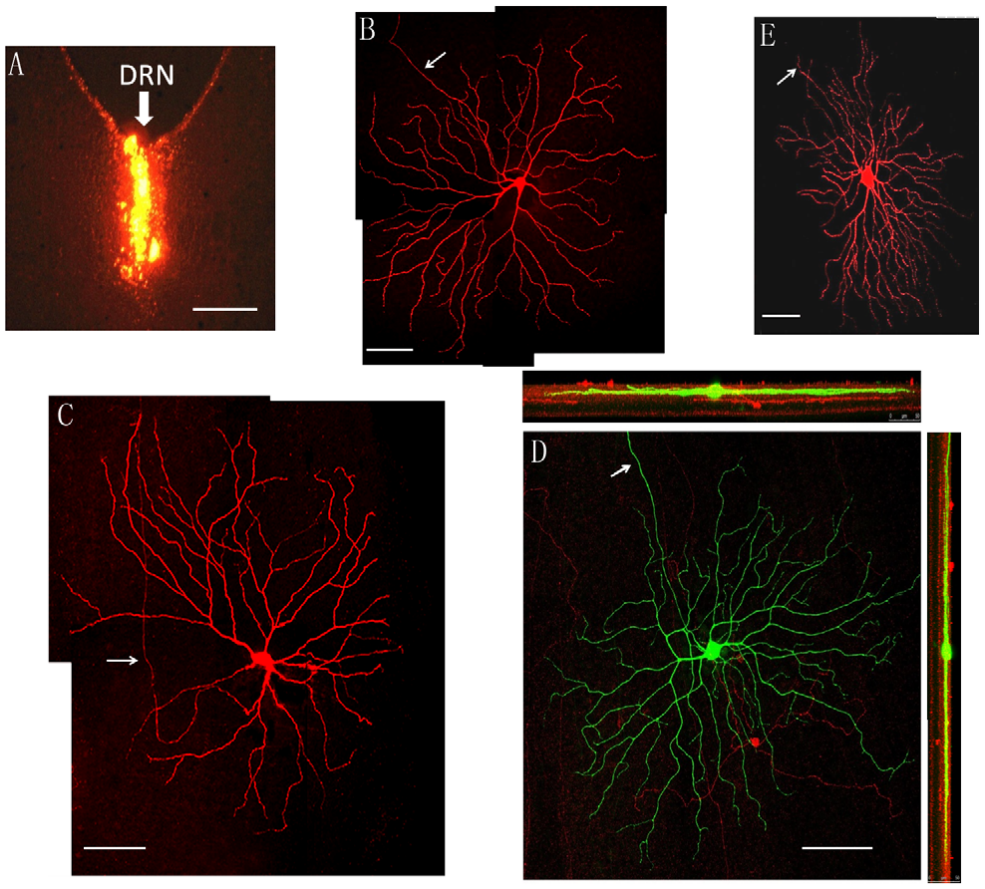
Dendritic morphology of DRN-projecting RGCs. (**A**) the DRN injection site. The arrow points to the injection site. (**B**)–(**D**) morphology of intracellularly injected DRN-projecting RGCs. (**B**) a large dendritic field alpha cell, (**C**) a relatively small dendritic field alpha cell, (**D**) a three dimensional reconstructed DRN-projecting alpha cell immunohistochemically stained with (FITC) and a melanopsin cell immunocytochemically stained for melanopsin (red); note lack of melanopsin immunoreactivity in DRN-projecting RGC. (**E**) morphology of a DRN-projecting non-alpha cell. The Arrows depict the axon. Scale bars: (**A**): 500 µm; (**B**): 200 µm; (**C**): 100 µm; (**D**): 100 µm; (**E**): 60 µm.

DRN-projecting RGCs were identified by retrograde transport of Microruby (8% biotinylated dextran-conjugated methylrhodamine) injected into the DRN of Mongolian gerbils (Meriones unguiculatus). A representative injection site is shown in Figure 1A. To analyze the morphology of DRN-projecting cells quantitatively, we intracellularly injected 104 DRN-projecting RGCs (n = 6 animals), 87 exhibited morphological characteristics similar to classic alpha cell morphology including large perikaryon (22±8 µm, n = 87), 3–4 primary dendrites, and large dendritic fields consisting of straight and radially oriented dendritic processes (398±143 µm, n = 87). Figures 1B–D illustrate three DRN-projecting cells that show typical alpha cell morphology. In addition, DRN-projecting RGCs can be further classified as ‘ON’ of ‘OFF’ depending on the sublamina of the interplexiform layer (IPL) in which their dendrites ramify; 71 of the 87 cells (80%) with alpha cell morphology stratified in the ON region of the IPL and 17 (20%) sent their dendrites into the OFF-sublamina. In addition to the alpha cells, a minority of DRN-projecting RGCs exhibited a completely different morphology; most of these cells had relatively small but dense dendritic fields (Fig. 1E). Since melanopsin expressing ipRGCs project to a broad range of central targets [14], [13], we examined the DRN-projecting RGCs for melanopsin protein. We were unable to detect melanopsin in any DRN-projecting alpha RGCs (or non-alpha DRN-projecting RGC) whereas melanopsin was readily detected in other RGCs (Fig. 1D).

### The distribution pattern of DRN-projecting ganglion cells

**Figure 2:**
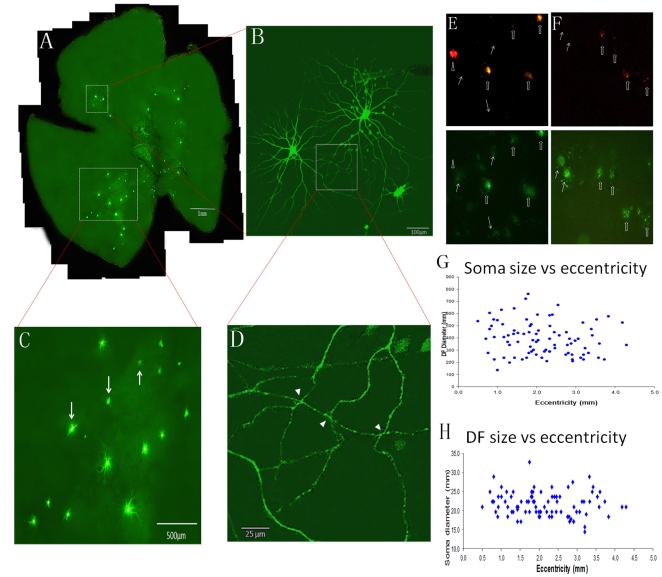
The distribution pattern of DRN-projecting RGCs. (**A**) the distribution pattern of DRN-projecting RGCs in a wholemount retina, (**B**) two neighboring alpha cells from the retina that have overlapped dendritic fields, (**C**) DRN-projecting alpha cells distributed in a small retinal area, (**D**) Higher-magnification view of overlapped dendritic fields of the two cells in (**B**), Arrow heads show possible synaptic contacts between the two, (**E**) double labeled RGCs innervate both DRN (red fluorescent staining) and dLGN (green fluorescent staining). (**F**) double labeled RGCs send their bifurcating axons to both DRN (red fluorescent staining) and SC (green fluorescent staining). The open arrows show RGCs projecting both nuclei and solid arrows illustrate RGCs project to dLGN or SC. The arrow head depicts a RGC that projects to DRN only. Dendritic field size and soma size variation with retinal eccentricity are provided in (**G**) and (**H**). Scale bars: A: 1 mm; B: 100 µm; C: 500 µm; D: 25 µm.

To estimate the distribution pattern of DRN-projecting alpha cells, we made one penetration at the DRN and delivered 1 µl of fluorescent microspheres to the injection site and filled every encountered retrogradely labeled cell; forty-seven cells were intracellularly injected in this retina (Fig. 2A). Many of the injected cells were found in the inferior retina and as shown in figure 2C, 11 of the 15 injected cells in this region were alpha cells, and they were evenly distributed in this area. As two of the neighboring alpha cells were studied in more detail (Fig. 2B), partly overlapped dendritic fields were observed (Fig. 2D). Figure 2D shows overlapping dendritic processes of the two cells under high magnification. It appears that the processes of the two cells make three possible synaptic contacts (indicated by white arrow heads, Fig. 2D). Although the nature of these contacts needs to be confirmed, electrical synaptic contacts (gap junctions) between alpha cells have been observed in adult mammalian retina [36]. To determine if the DRN-projecting RGCs also send axon collaterals to the dLGN [5], the primary thalamic visual nucleus that relays retinal signals to the visual cortex, a double-label experiment was carried out in a subset of animals. Red fluorescent microspheres were delivered to the DRN and green fluorescent microspheres were injected into the dLGN. Most of the DRN-projecting RGCs had bifurcating axonal projections to the dLGN. Although the number of double-labeled RGCs was not quantified, as shown in figure 2E, three out of four DRN-projecting RGCs in this selected field sent collaterals to the dLGN. The same approach was carried out to verify if SC, the major nucleus of the subcortical visual pathway, also receives bifurcating innervation from DRN-projecting ganglion cells. A similar projection pattern was observed in the SC. Four out of six SC-projecting cells sent their bifurcating axon to DRN (Fig. 2F). As in other rodents, neither soma size nor dendritic field diameter showed significant variation with retinal eccentricity (Fig. 2G,H).

### Visual response properties of DRN-projecting RGCs

**Figure 3:**
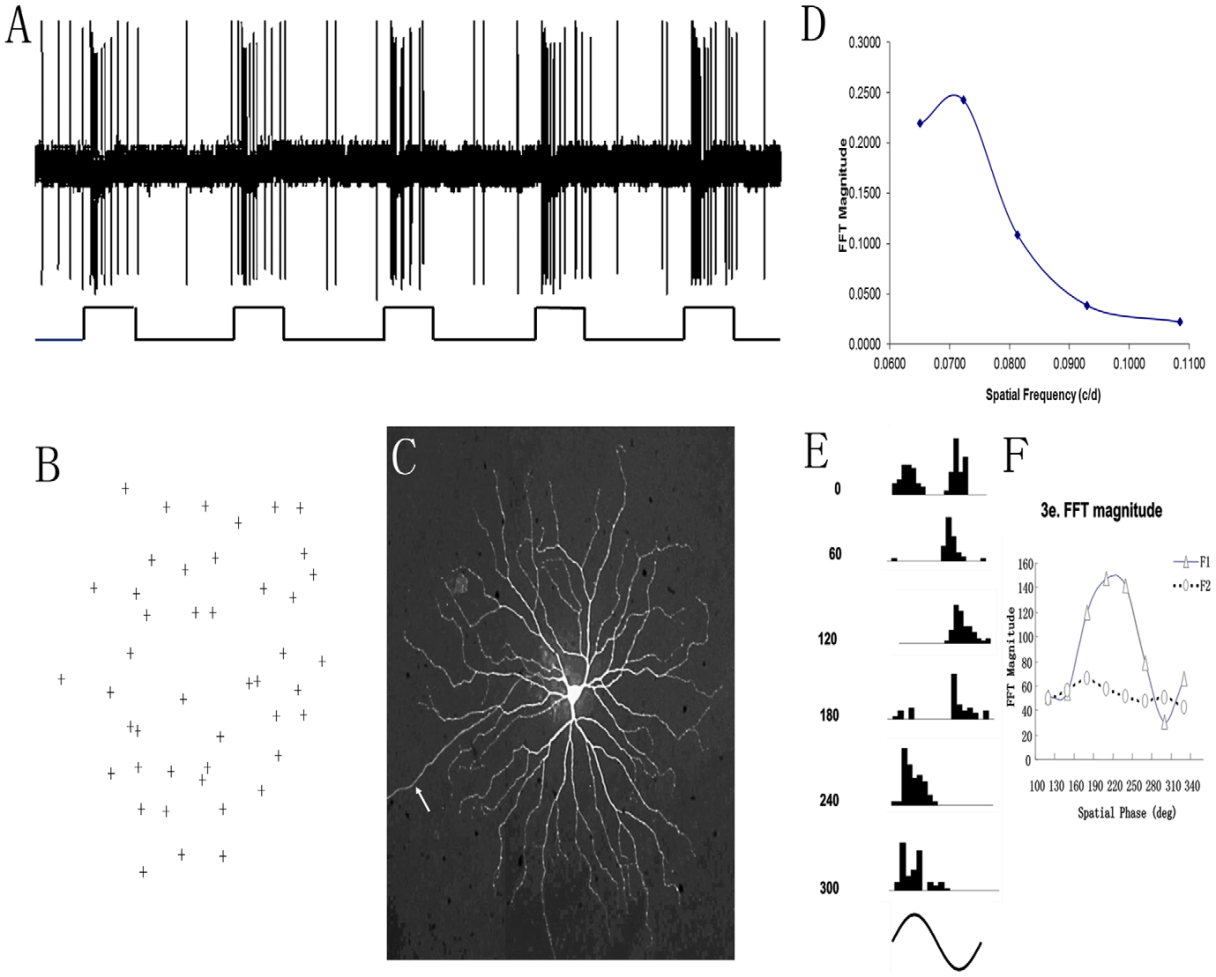
Physiological response properties of the DRN-projecting RGCs. (**A**) the visual response of an ON center Y-cell, (**B**) receptive field, and (**C**), dendritic morphology; the arrow reveals axon, (**D**) spatial frequency tuning of a Y-cell. Note the cell's response reached peak at 0.07 c/d. 3E presents the peristimulus-time histograms of the cell's responses to contrast reversal sinusoidal gratings (spatial frequency: 0.07 cyc/deg, temporal frequency: 2 Hz, contrast: 100%). The numbers to the left of the histograms depict spatial phases. This cell had frequency doubling at two spatial phases, 0° and 180°, respectively. 2F illustrates that, after fast Fourier transformation, the fundamental component (F1) is represented by triangular symbols (Δ) while the second harmonic (F2) is shown with open circular symbols (o).

Visual response properties of DRN-projecting RGCs were determined by recording from 51 retrogradely labeled DRN-projecting RGCs with large somas (alpha cells). All cells had typical Y cell transient response properties (ON = 34, OFF = 17). (Fig. 3A) shows the transient discharge pattern of an ON center Y-cell in response to a test light spot placed within the receptive field and (Fig. 3B) illustrates the plotted receptive filed for this Y-cell; the dendritic morphology of this same cell is alpha-like (Fig. 3C) consistent with the Y-type physiological responses. The spatial frequency response is shown in (Fig. 3D); the optimum spatial frequency was approximately 0.07 c/d. One of the classic visual response properties of Y-cells is their non-linear spatial summation [37], [24], [20]. (Fig. 3E) presents peristimulus-time histograms of a Y-cell in response to contrast reversal sinusoidal gratings (spatial frequency 0.07 c/d, contrast 100%). This cell had frequency doubling at two spatial phases, 0° and 180°, respectively. The fundamental component (F1) of the cell's discharges was drastically affected by spatial phases while the second harmonic (F2) was not (Fig. 3F), resembling the characteristics of Y cells.

### Are DRN-projecting Y cells intrinsically sensitive to light?

**Figure 4:**
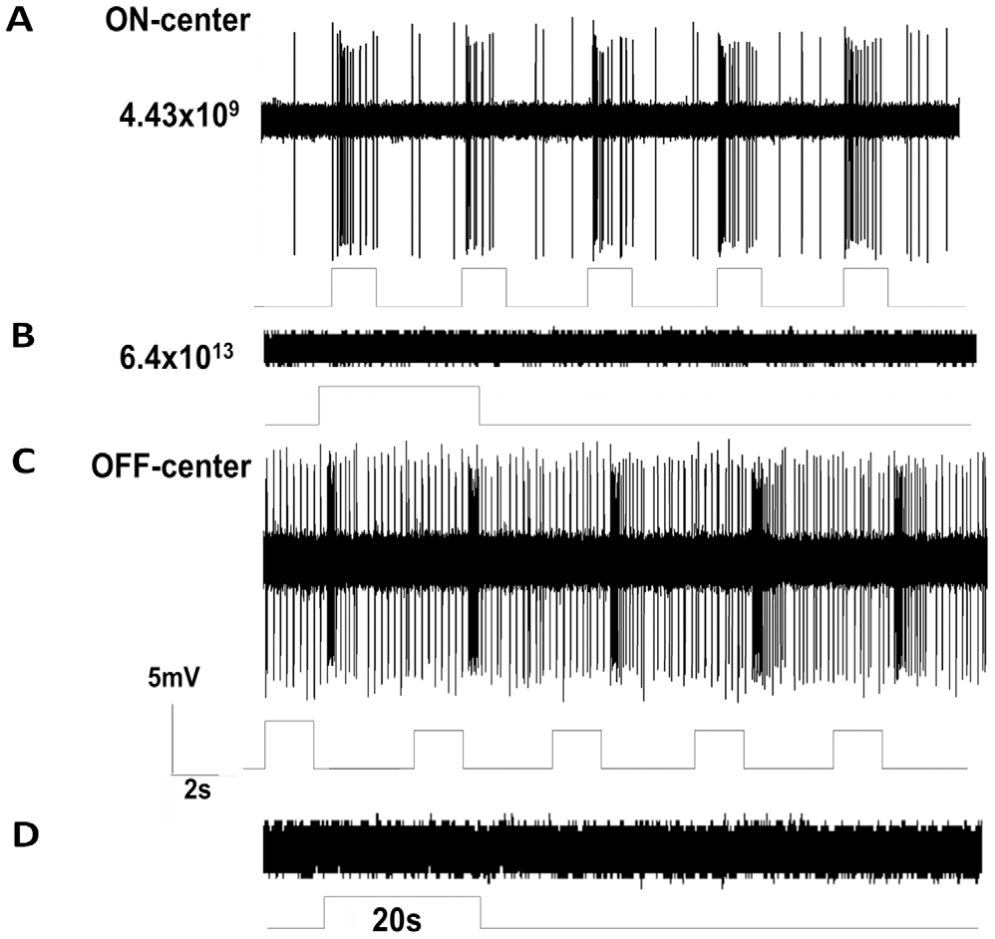
Visual response patterns of Y cells before and after synaptic transmission blockade. The synaptic blocker cocktail consisted of a mixture of excitatory and inhibitory neurotransmitter blockers including L-(+)-2-Amino-4-phosphonobutyric acid (AP-4, 100 µM), 6,7-Dinitroquinoxaline-2,3-dione (DNQX, 20 µM), DL-2-amino-5-phosphonovaleric acid (AP5, 50 µM), Picrotoxin (50 µM ) Strychnine (0.3 µM), and hexamethonimm bromide (200 µM) was added to the oxygenated Ames' medium. (**A**) visual responses of an ON center (upper) and (**C**) an OFF center (lower) DRN-projecting RGCs before synaptic transmission blockade. (**B**) and (**D**) response patterns of the cells after synaptic transmission blockade. The retinal irradiance of the stimuli was 6.4×10^13^ photons/cm^2^/sec, λ = 475 nm, and stimulation duration was 20 seconds.

To determine if DRN-projecting Y cells had any intrinsic response to light (some RGCs with alpha-like morphology have weak intrinsic photoresponses yet do not express detectable levels of melanopsin) [38], we recorded from DRN-projecting RGCs in the presence of a synaptic cocktail that removes all retinal synaptic transmission between photoreceptors and ganglion cells (n = 27). Among these cells, 18 were tested with the cocktail that also included cobalt chloride and 9 were tested without cobalt chloride. None of the DRN-projecting RGCs tested showed any sign of visual response after application of the cocktail with or without cobalt chloride. (Fig. 4) illustrates the discharge patterns of a representative pair of ON and OFF center DRN-projecting Y cells in response to a test light spot covering the entire center of their receptive fields (4.43×10^9^ photons/cm^2^/s) before (Fig. 4A,C) and after (Fig. 4B,D) the application of the synaptic blocker cocktail without cobalt chloride. As shown, both cells ceased to respond to the long duration, intense blue light stimulation after synaptic blocker application (20 sec, 6.3×10^13^ photons/cm^2^/s, λ = 475 nm).

## Discussion

The present study provides physiological and morphological evidence that the vast majority of RGCs innervating the DRN are Y (alpha) cells. It is demonstrated in numerous studies that alpha cells project to several different visual related nuclei indicating diversity in their function for visual perception. Until now, there is no evidence that links Y (alpha) cells with a non-imaging forming function. Therefore innervation of the DRN by Y (alpha) RGCs is both a novel observation and quite unexpected.

RGCs have been shown to innervate the DRN in several mammalian species including the cat [2], rat [3], [39], [4], Chilean degus [40], tree shrew [41], and mongolian gerbil [4], [5]. However, little is known about the morphology and physiology of RGCs projecting to DRN. Two early studies suggested that the DRN received direct retinal input, which consisted of a small number of RGCs [2], [3]. Fite and colleagues continued this line of work and reported a substantial number of DRN-projecting RGCs with both small and large somas and suggested that these cells arose from the non-image forming component of the retina [4], [5]. It was also reported that large soma DRN projecting cells also sent their bifurcating axon to dLGN [5]. Our results are in agreement with this finding. In addition, similar to alpha cells in others species; DRN-projecting RGCs also send their axons to the SC. Although, only a small number of DRN-projecting alpha cells were intracellularly stained in each retina, as shown in (Fig. 1D and 1G), the distribution of these stained cells appears to suggest a uniform pattern over the retina as reported in other rodent species [24]. The results show that 80% of DRN-projecting RGCs are Y(alpha)-like cells and the rest are small field RGCs. Additionally, we were unable to provide any evidence that DRN-projecting RGCs express melanopsin, consistent with a previous report [14].

The Y (alpha)-like RGC has been the focus of numerous structural and functional investigations over the last 50 years. To date, morphological evidence suggests that the alpha cell is well conserved in every mammal species examined [24]. Although the brisk-transient visual response pattern of this cell type is widely accepted [18], [19], [42], [21], there is evidence that ON- and some OFF-type Y-cells in the mouse retina exhibit a sustained discharge pattern [23]. However, as shown in (Fig. 3), under both scotopic and photopic conditions, Y (alpha) cells of the gerbil retina show clear transient visual response properties (Fig. 3A, 4A, 4C) and non-linear discharge patterns (Fig. 3E and Fig. 3F). The difference could be species related, and/or because the mouse is a nocturnal animal while the gerbil is diurnal. In line with our results, transient visual responses and a non-linear receptive field property is characteristic of Y-like cells in the primate, which is a diurnal species [21].

There is a wealth of information that Y (alpha) cells innervate visual related nuclei including the dLGN, SC, and pretectum [21], [25], [26], [27], [28], [29], [30], [31]. Alpha cells non-primate mammalian species or parasol cells in primates have been investigated extensively for many years regarding their roles in processing visual information [24], [20], [43], [21]. The large receptive field, transient discharge pattern, non-linear spatial summation, and achromatic luminance response properties of the alpha cells are believed to be fundamental for visual perception of motion and depth [44], [45], [46]. However, the role of Y-like pathway in the non-image forming processes has received little attention. Although functional roles of these DRN-projecting ganglion cells remains uncertain, there is evidence that DRN neurons respond to changes in the light and dark cycle [47], [48], [49], [50] and they are sensitive to phasic flashing light stimulation [51]. These results fit the visual response property of Y-cells. Since Y (alpha) like cells encode transient visual information and these cells consisted of major retinal inputs to the DRN, they could be responsible for conveying fast changing photic information to the DRN. On the other hand, the DRN is considered to be a part of the circadian entrainment circuit in mammals [52]. There is a direct anatomical connection between the DRN and SCN [39], [52]. Furthermore, in addition to the conventional retinohypothalamic tract (RHT) that provides luminance information necessary for entrainment, brief millisecond photo stimulation has been shown to be capable of inducing circadian phase shifts [53], [54], [55], [56]. Therefore, there could be a second pathway that conveys fast luminance changing signals to SCN. In line with this hypothesis, the present study suggests that Y (alpha)-like cells could play such a role in this pathway. Since the majority of RGCs that innervate DRN were Y (alpha) cells, these cells could play a major role in mediating non-visual function. To test this hypothesis, further studies should be carried out to determine specific roles of alpha cells in the non-imaging forming function.

A recent study reported that alpha-like cells in mouse retina express melanopsin [38] and therefore we sought to determine if the alpha cells described in the current study that are afferent to the DRN were melanopsin cells. However, we failed to detect melanopsin in alpha cells of the Mongolian gerbil nor were we able to evoke intrinsic light responses from these cells when all synaptic input was blocked (Fig. 4). Since the work of Ecker and colleagues (2010) was conducted on the mouse [38], a nocturnal animal while gerbil is diurnal, it is possible that this disparity is due to species difference.
